# Preimplantation genetic testing for hereditary hearing loss in Chinese population

**DOI:** 10.1007/s10815-023-02753-8

**Published:** 2023-04-05

**Authors:** Qingling Bi, Shasha Huang, Hui Wang, Xue Gao, Minyue Ma, Mingyu Han, Sijia Lu, Dongyang Kang, Aida Nourbakhsh, Denise Yan, Susan Blanton, Xuezhong Liu, Yongyi Yuan, Yuanqing Yao, Pu Dai

**Affiliations:** 1 College of Otolaryngology Head and Neck Surgery, Chinese PLA General Hospital, Chinese PLA Medical School, National Clinical Research Center for Otolaryngologic Diseases, Key Lab of Hearing Impairment Science of Ministry of Education, Key Lab of Hearing Impairment Prevention and Treatment of Beijing, #28 Fuxing Road, Beijing, 100853 China; 2 Departments of Otolaryngology Head & Neck Surgery, China-Japan Friendship Hospital, 2#Yinghua Road, Beijing, 100029 China; 3 Reproductive Center, Chinese PLA General Hospital, 28#Fuxing Road, Beijing, 100853 China; 4 Department of Otolaryngology, PLA Rocket Force Characteristic Medical Center, 16# XinWai Da Jie, Beijing, 100088 China; 5 Department of Clinical Research, Yikon Genomics, 1698 Wangyuan Road, Fengxian District Shanghai, 201400 China; 6 Department of Otolaryngology, University of Miami Miller School of Medicine, Miami, FL 33136 USA

**Keywords:** Hereditary hearing loss, PGT, Whole genome amplification, MALBAC, Genetic counseling

## Abstract

**Purpose:**

To evaluate the clinical validity of preimplantation genetic testing (PGT) to prevent hereditary hearing loss (HL) in Chinese population.

**Methods:**

A PGT procedure combining multiple annealing and looping-based amplification cycles (MALBAC) and single-nucleotide polymorphisms (SNPs) linkage analyses with a single low-depth next-generation sequencing run was implemented. Forty-three couples carried pathogenic variants in autosomal recessive non-syndromic HL genes, *GJB2* and *SLC26A4*, and four couples carried pathogenic variants in rare HL genes: *KCNQ4*, *PTPN11*, *PAX3*, and *USH2A* were enrolled.

**Results:**

Fifty-four in vitro fertilization (IVF) cycles were implemented, 340 blastocysts were cultured, and 303 (89.1%) of these received a definite diagnosis of a disease-causing variant testing, linkage analysis and chromosome screening. A clinical pregnancy of 38 implanted was achieved, and 34 babies were born with normal hearing. The live birth rate was 61.1%.

**Conclusions and relevance:**

In both the HL population and in hearing individuals at risk of giving birth to offspring with HL in China, there is a practical need for PGT. The whole genome amplification combined with NGS can simplify the PGT process, and the efficiency of PGT process can be improved by establishing a universal SNP bank of common disease-causing gene in particular regions and nationalities. This PGT procedure was demonstrated to be effective and lead to satisfactory clinical outcomes.

**Supplementary Information:**

The online version contains supplementary material available at 10.1007/s10815-023-02753-8.

## Introduction

Hearing loss (HL) is the third-largest cause of disability as reported by the Global Burden of Disease (2019). The report stated that 1.57 billion people had HL, which was defined as hearing loss in the better ear of over 20 decibels (dB) [1]. A population-based survey conducted in China in 2016 revealed 70 million individuals as having HL. The prevalence of disabling HL (loss in the better ear of more than 40 dB) was 0.85% in children from the provinces of Jilin, Gansu, Guangdong, and Shanxi, in the north, northwest, and southeast of China [2]. Disabling HL in childhood inhibits language development, educational achievement, and social-emotional development. Although hearing aids and cochlear implants are capable of restoring some auditory stimulation, these devices cannot restore natural hearing. Furthermore, the cost of hearing rehabilitation devices is a financial burden for many, and is not covered by insurance in developing countries.

The causes of HL include a variety of environmental factors, such as infection and ototoxic medicine; however, in developed countries, genetic factors account for 50–60% of childhood HL [3]. The World Health Organization (WHO) has recommended strategies for the prevention of HL due to maternal/infant infection and adverse events at the time of birth, and prompt initiation of habilitation for HL once diagnosed (https://www.who.int/news-room/fact-sheets/detail/deafness-and-hearing-loss). However, there is no effective and easily employed prevention strategy for hereditary HL yet.

Hereditary HL, a typically monogenic disorder, is highly heterogeneous, involving 124 known genes (https://hereditaryhearingloss.org/) and exhibiting all four Mendelian modes of inheritance. In Asia, Europe, and America, 35~60% of HL patients were identified as having a genetic etiology through targeted genomic enrichment with massively parallel sequencing [4–6]. Genetic testing cannot only identify a precise molecular etiology but can also serve as the basis of prenatal diagnosis (PND) for patients with hereditary diseases to prevent recurrence. Preimplantation genetic diagnosis, more recently described as preimplantation genetic testing for monogenic disease (PGT-M) [7], first appeared in 1990 [8]. PGT-M is an effective means of preventing birth defects associated with hereditary disorders where the genetic etiology can be confirmed [7]. However, attitudes towards PND and PGT for non-lethal disorders such as HL vary greatly depending upon the culture, financial status, and religion of the at-risk individuals, as well as government policies. These factors, combined with the complex technical processes, have resulted in fewer PGT cases with HL being reported (Table S1) [9–17] than are in cases of another monogenic disease such as Huntington disease, cystic fibrosis, and neurofibromatosis.

In China, approximately 80% of HL cases diagnosed as hereditary HL have been shown to be autosomal recessive genetic, with both parents having normal hearing and carrying a single copy of the altered gene [18]. Thus, the prevention of hereditary HL is targeted not only at families with multi-generational histories of HL but also toward at-risk carrier families. The purpose of this study was to develop an efficient PGT strategy to prevent births with HL in at-risk families.

## Materials and methods

### Patients’ recruitment

Forty-seven couples were recruited from the Genetic Testing Center for Deafness, Chinese PLA General Hospital between October 2016 and July 2020. Thirty-seven normal hearing couples are carrier-carrier pattern; they have already had HL children. Eight couples are patient-carrier pattern. One carrier couple was detected by a proactive pre-pregnancy genetic testing. One carrier couple was detected by neonatal genetic screening; their first child was a HL gene carrier. All participants desired to have children with normal hearing and underwent face-to-face genetic counseling along with their immediate family members.

### Variants interpretation

All the variants identified in the study participants were assessed in detail (Table 1). Variants were classified according to the American College of Medical Genetics (ACMG) guidelines [19], and variant information was retrieved from the ClinVar (https://www.ncbi.nlm.nih.gov/clinvar/) and Deafness Variation Database (http://deafnessvariationdatabase.org/) websites.

**Table 1:** Pathogenicity analysis of the variants in PGT

	Nucleotide change	Amino acid change	Heterozygous numbers	Homozygous numbers	Allele frequency in patients	Homozygous or Compound heterozygous in Gnom AD	Variants type	ACMG/AMP variant classification	Clinical significance (Clinic Var)	Clin Var Accession	HL variation database
*GJB2*	c.235delC	p.Leu79Cysfs*3	444	428	13.00%	None	Missense	PS3+PS4+PM1+PM3+PM4+PP3	Pathogenic	VCV000017014	Pathogenic
	c.299delAT	p.His100Argfs*14	254	28	3.10%	None	Frameshift	PS3+PS4+PM1+PM3+PM4+PP3	Likely pathogenic	VCV000633242	Pathogenic
	c.109A>G	p. Val37Ile	65	44	1.53%	None	Missense	PS3+PM1+PM3+PP3	Pathogenic	VCV000017023	Pathogenic
	c.512insAACG	p.Trp172Thrfs*40	53	0	0.53%	None	Frameshift	PS3+PS4+PM1+PM3+PM4+PP3	-	-	Pathogenic
	c.257C>G	p. Thr86Arg	40	0	0.40%	None	Missense	PS4+PM1+PM3+PP3	Pathogenic	VCV000631697	Pathogenic
	c.605ins46	p. Cys202*	20	0	0.20%	None	Missense	PS3+PS4+PM1+PM3+PM4+PP3	Pathogenic	VCV000627447	Pathogenic
*SLC26A4*	c.919-2A>G	splicing site	501	293	10.87%	None	Splice acceptor	PS3+PS4+PM1+PM3+PP3	Pathogenic	VCV000004840	Pathogenic
	c.2168A>G	p.His723Arg	194	12	2.18%	None	Missense	PS4+PM1+PM3+PP3	-	-	Pathogenic
	c.1174A>T	p.Asn392Tyr	74	3	0.80%	None	Missense	PS4+PM1+PM3+PP3	Pathogenic	VCV000446453	Pathogenic
	c.1226G>A	p.Arg409His	56	3	0.62%	None	Missense	PS4+PM1+PM3+PP3	Likely pathogenic	VCV000043497	Pathogenic
	c.1229C>T	p.Thr410Met	48	3	0.54%	None	Missense	PS4+PM1+PM3+PP3	Pathogenic	VCV000043498	Pathogenic
	c.1975G>C	p.Val659Leu	43	0	0.43%	None	Missense	PS4+PM1+PM3+PP3	Pathogenic/Likely pathogenic	VCV000188889	Pathogenic
	c.589G>A	p.Gly197Arg	37	0	0.37%	None	Missense	PS4+PM1+PM3+PP3	Likely pathogenic	VCV000043562	Pathogenic
	c.754T>C	p.Ser252Pro	7	0	0.07%	None	Missense	PS4+PM1+PM3+PP3	-	-	Pathogenic
	c.1586T>G	p.Ile529Ser	5	0	0.05%	None	Missense	PS4+PM1+PM3+PP3	Pathogenic/Likely pathogenic	VCV000189160	Pathogenic
	c.249G>A	p.Trp83*	2	0	0.02%	None	Stop gained	PS4+PM1+PM3+PM4+PP3	Pathogenic	VCV000370650	Pathogenic
	c.2014G>A	p.Gly672Arg	1	0	0.01%	None	Missense	PS4+PM1+PM3+PP3	-	-	Likely pathogenic
	c.697G>C	p.Val233Leu	1	0	0.01%	None	Missense	PS4+PM1+PM3+PP3	Uncertain significance	VCV000043564	Pathogenic
	c.917insG	p.Val306Glyfs*24	1	0	0.01%	None	Frameshift	PS4+PM1+PM3+PM4+PP3	Pathogenic	VCV000004815	Pathogenic
	c.1373T>C	p.Leu458Pro	1	0	0.01%	None	Missense	PS4+PM1+PM3+PP3	-	-	Pathogenic
	c.1286C>A	p.Ala429Glu	1	0	0.01%	None	Missense	PS4+PM1+PM3+PP3	-	-	Pathogenic
	c.1540C>T	p.Gln514Lys	1	0	0.01%	None	Missense	PS4+PM1+PM3+PP3	Pathogenic	VCV000004841	Pathogenic
	c.1522A>G	p.Thr508Ala	1	0	0.01%	None	Missense	PS4+PM1+PM3+PP3	Pathogenic	VCV000179732	Pathogenic
	c.439A>G	p.Met147Val	1	0	0.01%	None	Missense	PS4+PM1+PM3+PP3	Pathogenic	VCV000691508	Pathogenic
	c.281C>T	p.Thr94Ile	1	0	0.01%	None	Missense	PS4+PM1+PM3+PP3	Pathogenic	VCV000371034	Pathogenic
	exon1-3del	CNV	1	0	0.01%	None	Exon deletion	PS4+PM1+PM3+PM4+PP3	-	-	-
	exon5-6 del	CNV	2	0	0.02%	None	Exon deletion	PS4+PM1+PM3+PM4+PP3	-	-	-
	Nucleotide change	Amino acid change	AD/AR	Allele frequency in Gnom AD			Variants type	ACMG/AMP variant classification	Clinical significance (Clinic Var)	Clin Var accession	HL variation database
*PTPN11*	c.1510A>G	p.Met504Val	AD	0.0000			Missense	PS3+PS4+PM1+PM2+PP1+PP2	Pathogenic	VCV000040562	-
*KCNQ4*	c.733G>A	p.Gly245Arg	AD	0.0000			Missense	PS3+PS4+PM1+PM2+PP1+PP2 +PP3	-	-	VUS
*PAX3*	c.210C>A	p.Cys70*	AD	-			Nonsense	PS4+PM1+PM2+PP1++PP2+PP3	Likely pathogenic	VCV000547732	-
*USH2A*	c.4576G>A	p.Gly1526Arg	AR	-			Missense	PS4+PM1+PM2+PM4+PP1+PP2 +PP3	-	-	Pathogenic
	c.99insT	p.Arg34Serfs*41		0.00001			Frameshift	PS1+PS4+ PM1+PM2+PM3+ PM4+PP3	Pathogenic	VCV000520636	Pathogenic

*CNV* Copy number variation; *AD* autosomal dominant; *AR* autosomal recessive; *NSHL* nonsyndromic hearing loss; *VUS* variant of uncertain significance

### Clinical PGT procedure

The PGT-M procedure combined multiple annealing and looping based amplification cycles (MALBAC) and single-nucleotide polymorphisms (SNPs) linkage analyses with a single low-depth next-generation sequencing run, which was developed base on MASALA [20] (mutated allele revealed by sequencing with aneuploidy and linkage analyses).

#### Embryo biopsy

Embryo biopsy procedures were performed at the Reproductive Medicine Center of Chinese PLA General Hospital as described in a previous article [21]. Intracytoplasmic sperm injection (ICSI) was performed to avoid surplus sperm contamination. All embryos were biopsied at the blastocyst stage; three to five trophectoderm cells were biopsied from the expanding blastocysts using a rubbing dissection method on day 5.

#### Whole-genome amplification by MALBAC and genetic analysis

Cell lysis and whole-genome amplification (WGA) were performed using a MALBAC single-cell WGA kit (Yikon Genomics Inc., catalog number: YK001A/B) according to the manufacturer’s protocol.

#### Variant site detection and linkage analysis

Ninety SNP markers linked to alleles with pathogenic variants were selected for each gene within 1 Mb upstream or downstream of the gene. The frequencies of the major alleles were greater than 0.1 in the Asian population. We performed multiplex PCR using the MALBAC amplification product as a template with 90 pairs of SNP-specific primers in each PCR. The variant sites were amplified in individual PCRs using specific primers. These PCR products were purified, subjected to library construction, and then sequenced on the Illumina HiSeq 2500 platform (1 M reads). The Genome Analysis Toolkit, version 3.5, Best Practices workflow was used to identify variants in the PCR-amplified regions. SNPs with less than 100× coverage were defined as “low depth”. Sanger sequencing was used to further confirm the detection of variants.

#### CNVs analysis

The MALBAC-amplified products were purified and sequenced on the Illumina HiSeq 2500 platform (1.5 M reads). Uniquely mapped reads were extracted from the alignment reads (bam file). The entire reference genome was divided into non-overlapping observation windows (bins), each 1 Mb in size. The read numbers and GC content were calculated for each bin. GC bias correction was applied for every 1% of GC content. R software (version 3.0.0) was used to graphically display the GC-corrected relative read numbers of each bin for the visualization of CNVs.

#### Transfer of embryos and pregnancy follow-up

**Fig. 1:**
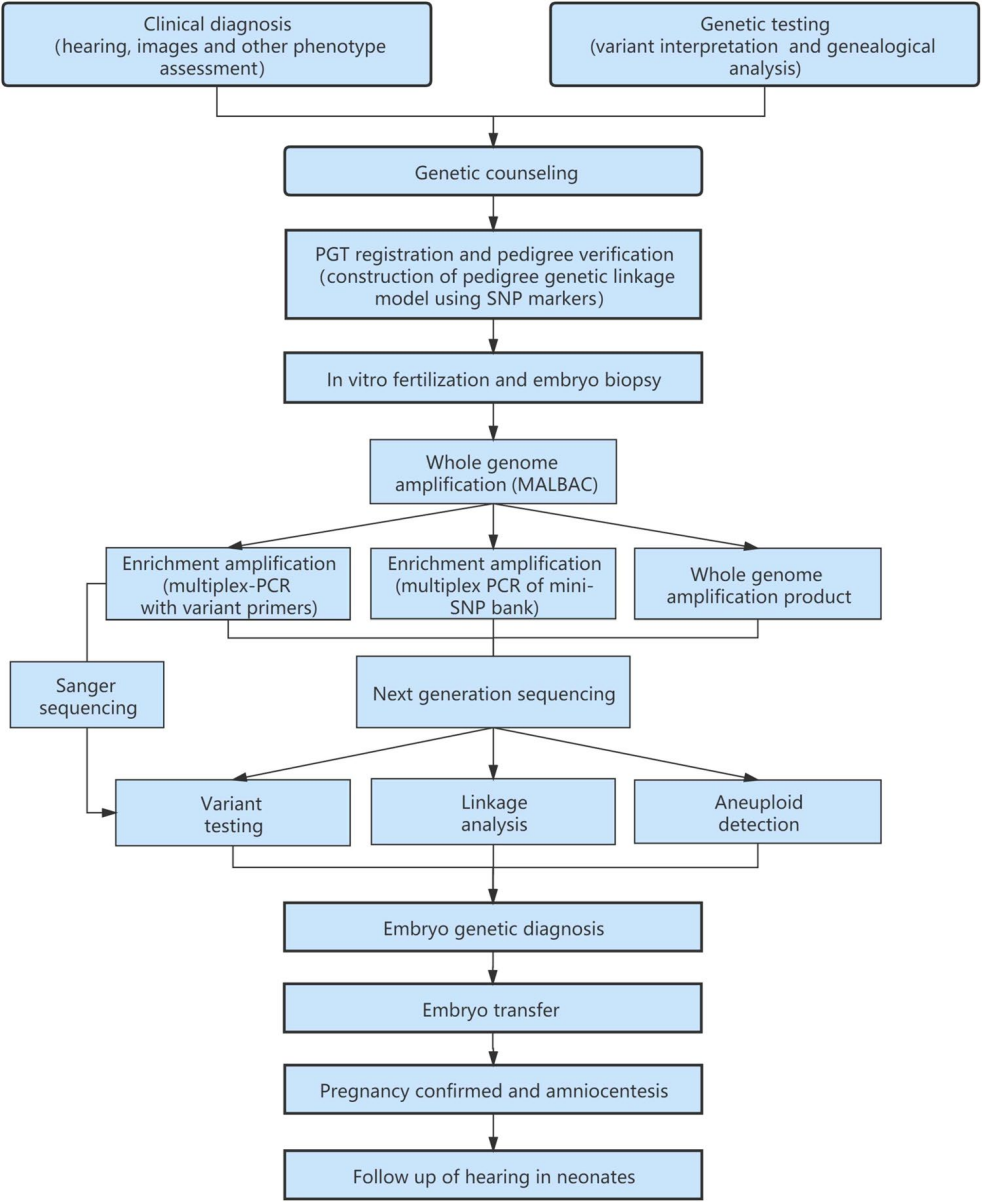
PGT clinical procedure and testing workflow. Trophectoderm cell lysis and WGA were performed using a MALBAC single-cell WGA kit. Enrichment PCR amplification of the WGA products was conducted using SNPs- and variant-specific primers. The PCR products were then sequenced by NGS; linkage analysis, chromosome aneuploidy analysis, and variant diagnosis were carried out simultaneously

The first transfer was performed 2–3 months after ICSI via artificial or natural cycles. After the exclusion of aneuploid, affected, or undetermined embryos, one or two unaffected embryos were transferred. The human chorionic gonadotropin test was performed on day 10 following transfer. Pregnancy was confirmed by ultrasound at 8 weeks’ gestation. Clinical pregnancy was defined as the observation of fetal heartbeat. Prenatal diagnosis was performed by amniocentesis at 20 weeks gestation. Auditory brainstem response, otoacoustic emissions, and gene detection in cord blood were performed following birth. The clinical PGT procedure and testing workflow are shown in Fig. 1.

The study was approved by the Research Ethics Committee of the Chinese PLA General Hospital (approval number S2019-203-01). Informed written consent was obtained from all patients or their guardians prior to genetic analysis and PGT.

## Results

Forty-seven couples were enrolled in the PGT-M with PGT-A procedure. The ages of the female participants undergoing PGT-M ranged from 25 to 40 years, with a mean age of 32.3 years. Forty-three couples carried pathogenic variants in autosomal recessive non-syndromic HL genes, *GJB2* and *SLC26A4*, and one case carried a variant in the autosomal dominant non-syndromic HL gene, *KCNQ4*. Three cases were diagnosed with syndromic HL, including one case of Noonan syndrome (*PTPN11*), one case of Waardenburg syndrome (*PAX3*), and one case of usher syndrome (*USH2A*).

The haplotypes were constructed using heterozygous SNPs from the couples and their existing children with HL. However, in the case of two couples (F15421, F1884), they had no child, and as such the haplotypes was constructed from the couples and their parents. Genotyping of 90 SNPs in the proximity (1 Mb upstream or downstream) of each gene included in the PGT procedure led to the identification of between 14 and 54 informative heterozygous SNPs for each family that were subsequently used in linkage analyses.

A total of 54 ICSI cycles were implemented on 47 couples, 40 (out of 47; 85.1%) of which underwent one cycle and the remaining 7 (14.9%) underwent two cycles. A total of 340 embryos were cultured and biopsied. One hundred and fourteen (33.5%) euploid embryos were found to be either without pathogenic variants or with a heterozygous pathogenic variant only involving autosomal recessive genes. Fifty-nine transfers were completed, and a clinical pregnancy was achieved in 38 cases, with a clinical pregnancy rate of 64.4%. Four miscarriages (6.8%) occurred in early pregnancy. We performed the chromosome analysis on the abortion tissues in 3 cases. A del (7) (q11.21q11.23) mosaicism was detected in one case, which was inconsistent with the embryo diagnosis result. We are not sure if it was a PGT-A error or a mosaic embryo. The other two cases were euploids, and we assumed the cause of the miscarriage was not due to embryonic factors. The abortion tissue analysis was not carried out in case F8241. One intrauterine death happened in the 38th week due to torsion of the umbilical cord. Thirty-four babies were born with normal hearing, including a set of twins, with a cumulative live birth rate of 61.1% (33 live births/54 cycles). Five couples still have embryos available and are waiting for transfer. Eight couples (17.0%) withdrew from the procedure due to unsuccessful pregnancy in one or two cycles. The auditory brainstem response and otoacoustic emission measurements were normal for all neonates. During the 6- to 52-month follow-up, no infant developed HL or any other developmental abnormalities. The details of each cycle are presented in Table 2.

**Table 2:** Details of the at-risk families enrolled in the PGT process and their clinical outcomes

Family ID	Female age	Gene	Inheritance mode	Maternal variants	Paternal variants	Cycles	Total embryos	Transferable	Implantation times	Clinical pregnancy	Outcome
F6711	32	*SLC26A4*	AR	c.1975 G>C	c.1174A>T	1	17	11	2	1^st^ N,2^nd^Y	Normal hearing (Twins)
F6166	32	*SLC26A4*	AR	c.1174A>T	c.919-2A>G	1	9	3	2	1^st^Y※ 2^nd^Y	Normal hearing
F3497	36	*SLC26A4*	AR	c.1229C>T	c.2168A>G	1	9	2	1	Y	Normal hearing
F1884	26	*GJB2*	AR	c.299delAT	c.235delC	2	11	3	2	1^st^ N,2^nd^ N	Withdrew
F7414	27	*SLC26A4*	AR	c.2168A>G	c.919-2A>G	1	5	3	1	Y	Normal hearing
F8659	30	*SLC26A4*	AR	c.919-2A>G	c.919-2A>G	1	6	3	1	Y	Normal hearing
F8085	33	*SLC26A4*	AR	c.919-2A>G	c.919-2A>G/c.1225C>T	1	5	2	2	1^st^Y*,2^nd^ Y	Normal hearing
F10284	31	*SLC26A4*	AR	c.2168A>G	c.697 G>C	1	7	0	0	/	Withdrew
F7047	32	*GJB2*	AR	c.257C>G	c.605ins46	1	3	1	0	/	Waiting for transfer
F2530	35	*SLC26A4*	AR	exon 5-6 deletion	c.919-2A>G	1	9	6	1	Y	Normal hearing
F8241	32	*SLC26A4*	AR	c.589G>A	c.1229C>T	2	13	3	3	1^st^ N,2^nd^ Y* 3^rd^ Y	Normal hearing
F10449	32	*GJB2*	AR	c.235delC	c.235delC	1	6	2	2	1^st^N,2^nd^ Y	Normal hearing
F12131	33	*GJB2*	AR	c.235delC	c.235delC	1	6	3	1	N	Waiting for transfer
F12198	31	*GJB2*	AR	c.512insAACG	c.109G>A/c.235delC	1	2	1	1	Y	Normal hearing
F8096	40	*SLC26A4*	AR	c.1975G>C	c.2168A>G	1	3	1	1	Y	Normal hearing
F932	37	*SLC26A4*	AR	c.919-2A>G	c.1975G>C	2	16	4	2	1^st^ Y*2^nd^ Y	Normal hearing
F12244	31	*SLC26A4*	AR	c.754T>C	c.2168A>G	1	13	2	1	Y	Normal hearing
F9323	33	*GJB2*	AR	c.299delAT	c.299delAT	1	7	2	1	N	Waiting for transfer
F14118	29	*SLC26A4*	AR	c.1586T>G	c.249G>A	1	10	3	3	1^st^ Y*2^nd^ N 3^rd^ N	Withdrew
M464	28	*SLC26A4*	AR	exon5-6 deletion	c.919-2 A>G	1	4	1	1	Y	Normal hearing
F308	33	*SLC26A4*	AR	c.754T>C	c.2014G>A	1	10	6	1	Y	Normal hearing
F12585	39	*GJB2*	AR	c.235delC	c.235delC	1	2	0	0	/	Withdrew
F3105	38	*SLC26A4*	AR	c.919-2 A>G	c.919-2 A>G	1	7	0	0	/	Withdrew
F6968	32	*GJB2*	AR	c.512insAACG	c.235delC	1	4	2	1	N	Waiting for transfer
F2242	36	*SLC26A4*	AR	c.919-2 A>G	c.1226G>A	1	4	1	1	N	Withdrew
F7105	35	*SLC26A4*	AR	c.919-2 A>G	c.1522A>G	1	2	1	1	N	Withdrew
F14171	38	*GJB2*	AR	c.235delC	c.235delC	1	6	1	1	Y	Normal hearing
F10717	32	*GJB2*	AR	c.235delC	c.109G>A	1	8	2	1	Y	Normal hearing
F11595	32	*SLC26A4*	AR	c.1540C>T	c.919-2A>G	1	12	4	3	1^st^ N,2^nd^ N 3^rd^ Y	Normal hearing
F15108*	29	*GJB2*	AR	c.235delC	c.235delC	2	5	1	1	Y	Normal hearing
F14048	28	*GJB2*	AR	c.299delAT(homo)	c.109G>A	1	4	1	1	Y	Normal hearing
F13373	36	*GJB2*	AR	c.235delC	c.299delAT	2	9	3	1	Y	Normal hearing
F15782	32	*SLC26A4*	AR	c.1373T>C	c.919-2A>G	1	3	1	1	Y	Normal hearing
F11968	31	*SLC26A4*	AR	c.919-2 A>G	c.281C>T	1	1	1	1	N	Withdrew
M759	27	*GJB2*	AR	c.235delC	c.176del16/c.299delAT	2	9	3	2	1^st^N,2^nd^ Y	Normal hearing
F8007	38	*GJB2*	AR	c.235delC	c.235delC	1	6	2	2	1^st^ N,2^nd^ Y	Normal hearing
F15421*	25	*GJB2*	AR	c.235delC	c.176del16/c.235delC	1	4	3	1	Y	Normal hearing
F16250	29	*SLC26A4*	AR	c.917insG/c.919-2A>G	c.2168A>G	1	8	1	1	Y	Normal hearing
F16178	27	*SLC26A4*	AR	c.919-2 A>G	c.1286C>A	1	11	5	2	1^st^N,2^nd^ Y	Normal hearing
M1318	33	*SLC26A4*	AR	exon1-3 deletion	c.1174A>T	1	6	5	1	N	Normal hearing
M784	33	*SLC26A4*	AR	exon5-6 deletion	c.1519delT	1	1	1	1	Y	Normal hearing
F17172	33	*GJB2*	AR	c.235delC	c.235delC	1	4	1	1	Y	Normal hearing
M855	35	*SLC26A4*	AR	c.439A>G	c.2168A>G	1	10	1	0	-	Waiting for transfer
M651	25	*PTPN11*	AD	-	c.1510A>G	1	11	3	2	1^st^ N,2^nd^ Y	Normal hearing
M75	35	*KCNQ4*	AD	c.733G>A	-	2	20	2	1	Y	Normal hearing
M401	30	*PAX3*	AD	c.210C>A	-	1	2	2	1	Y	Normal hearing
M1026	35	*USH2A*	AR	c.4576G>A	c.99insT	1	10	5	2	1^st^ N,2^nd^ Y	Normal hearing
Total 47	Mean age 32.3					54	340	114	59	38(38/59，64.4%)	Live births 33 (33/54,61.1%)

*AR* Autosomal recessive; *AD* autosomal dominant; *Y* pregnancy; *N* no pregnancy; ^/^no implantation; ^*^miscarriage; ^※^intrauterine death in the 38th week

In our study group, 44 couples were identified as carriers of autosomal recessive HL gene, *GJB2*, *SLC26A4*, and *USH2A*. One hundred and seven transferable embryos were screened out, including 40 (40/107, 37.4%) wild type and 67 (67/107, 62.6%) carrier embryos. Eighteen (18/44, 40.9%) couples did not get any wild type embryo, and they chose to transfer the carrier embryos, and 4 (4/44, 9.1%) couples chose to transfer carrier embryos after wild type embryos were used up. No couples refused to transfer a carrier embryo in our study. Details were described in Table S2.

**Fig. 2:**
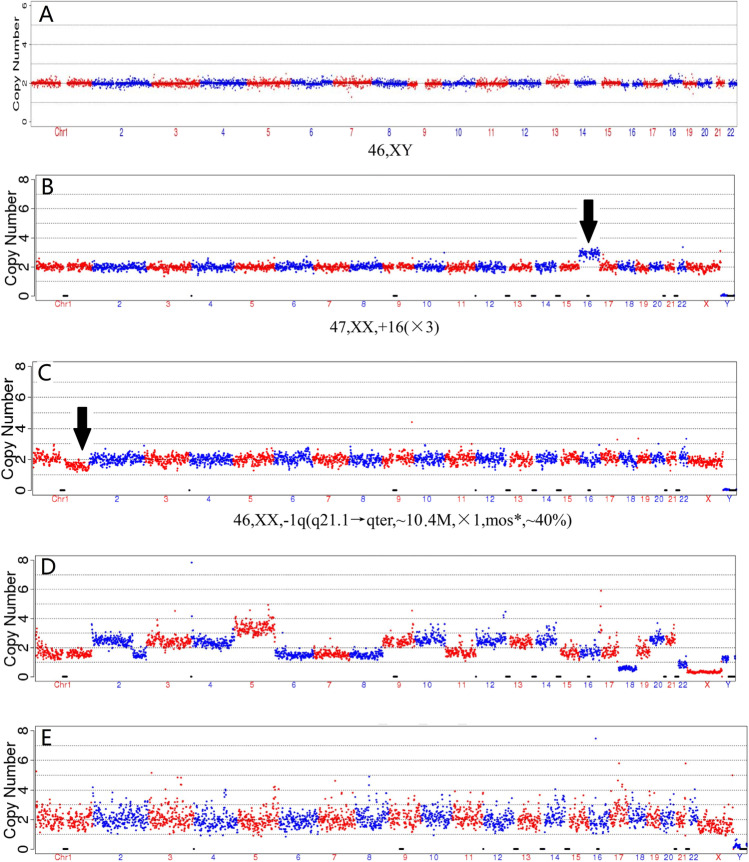
Preimplantation genetic testing for aneuploidy (PGT-A) result samples: **A** Chromosome euploid; **B** trisomy 16; **C** del1q21.1qter mosaicism; **D** multi-chromosome abnormality; **E** contamination

Three hundred and three (303/340, 89.1%) embryos were given a definite diagnosis as a result of the disease-causing variant testing, linkage analysis, and preimplantation genetic testing for aneuploidy (PGT-A) (Fig. 2A–E). Any CNVs greater than 4 Mb were reported, and 130 (130/340, 38.2%) embryos were identified as aneuploid (Fig. 2B, C). The difference in the aneuploid ratio between cycles ranged from 0% to 100% (Median 42.9%). There was no significant association between the aneuploid ratio and the women age at implant (Mann–Whitney test *U*=207.5, *p*=0.59).

The diagnostic results were inconclusive in 37 (37/340, 10.9%) embryos. MALBAC failure was detected in three (3/340, 0.9%) embryo cell samples upon quality control testing. For 337 embryos, 501 rounds of PCR and Sanger sequencing were carried out successfully, and 14 ADOs were identified with an ADO rate of 2.8% (14/501). In 10 cases, the ADO was identified through linkage analysis. In the other 4 cases, the non-mutant allele dropped out, and these did not affect the diagnosis. For instance, in one case (F10284) embryo number 4, we detected a homozygotic mutation of *SCL26A4 c.2168A>G*, but the maternal and paternal mutations were *SCL26A4 c.2168A>G* and *SCL26A4 c.697G>C*. It was clear that the non-mutant allele had dropped out, and as such, when taken in combination with the result of the linkage analysis, the diagnosis was clear. The CNV detection results of 9 embryos showed multiple chromosomal abnormalities (Fig. 2D). In these cases, the biopsy material was assumed to have contained broken cells. For 4 embryos, much of the sequencing data did not match the reference genome due to loss or contamination of biopsy cells (Fig. 2E). Variant or SNP sequencing failures of unknown cause occurred in 11 cases, also leading to inconclusive diagnoses.

## Discussion

HL is the most common neurosensory disorder worldwide [22]. With the development of next-generation sequencing (NGS) techniques, hereditary HL can easily be detected at the molecular level, heralding the arrival of the era of precision medicine. A recent etiological study in China showed that the use of next generation sequencing (NGS) to detect 129 HL genes led to 52.19% diagnostic rate in sporadic HL patients (*n* = 433), and 56.67% in HL pedigrees (*n* = 78) [18]. Meanwhile, with the implementation of concurrent newborn hearing and genetic screening [23, 24], large numbers of carriers in normal hearing population can be identified in advance. Our recent study reported that among 180,469 newborns in Beijing, China, the carrier rate of the nine major pathogenic variants across four genes (*GJB2*, *GJB3*, *SLC26A4*, and mtDNA *12S rRNA*) was 4.5% [23]. With the context of genetic epidemiology, the three-level prevention of hereditary hearing loss has been conceived and implemented in China over the years. Briefly, the primary prevention includes genetic screening before pregnancy and PGT-M; Secondary prevention includes the detection of deafness genes in pregnant women and the prenatal diagnosis; The tertiary prevention includes the joint screening of hearing and genes in newborns, as well as the genetic diagnosis and hearing rehabilitation of deaf patients. Therefore, in addition to those families who have already had HL members, many at-risk families with carriers were emerging by pre-pregnancy genetic screening and neonatal genetic screening. Both kind of conditions were enrolled in our PGT-M procedure. Prospectively, PGT-M procedure with great efficiency should be applied to a wider population.

The gene and variant spectra of hereditary HL vary greatly according to race. In a mixed-race genetic study from the USA with 1119 cases [6], nearly 75% of the genetic diagnoses were attributable to 10 HL genes, with *GJB2* being the most common and accounting for the etiology in 22% of the diagnosed patients. The next three most-frequently implicated genes were *STRC* (16%), *SLC26A4* (7%), and *TECTA* (5%). *GJB2* and *STRC* are the most common HL genes in Caucasians as well as in Hispanics. The Middle East has a high rate of consanguineous marriage, thereby increasing the risk of recurrent hereditary HL. *GJB2*, *SLC26A4*, *MYO15A*, and *TMC1* are the most prevalent genes involved in non-syndromic HL in the Middle East (Iran, Turkey, and Pakistan) [25]. In sub-Saharan Africa, the etiologic genetic diagnosis rate is 4% for certain specific genes (*MYO7A*, *MYO6*, *SLC26A4*, *SIX1*, *TRIPBP*, and *POU3F4*) [26]. According to the previous study [18], *GJB2* and *SLC26A4* were the predominant etiologies of HL in the Chinese population with HL (43.5% of *GJB2* and *SLC26A4* vs. 8.7% of other rare HL genes). The pathogenicity of hotspot mutations in these two genes is well defined. Thus, a PGT-M strategy target to *GJB2* and *SLC26A4* should covered over 80% genetic diagnosed at-risk families.

Prenatal diagnosis plays an important role in preventing congenital disabilities caused by severe genetic diseases. A quantitative circulating single-molecule amplification and resequencing technology cSMART assay was developed for the non-invasive prenatal diagnosis (NIPD) of autosomal recessive non-syndromic HL caused by *GJB2* and *SLC26A4* pathogenic variants [27]. Zhang et al. [28] implemented a novel NIPD approach for de novo mutations in causative genes implicated in common dominant monogenic diseases including HL. Surveys in developed countries have shown that people often undergo PND to inform the use of a neonatal early hearing habilitation plan, and are unlikely to terminate the pregnancy [29, 30]. However, also in developing countries, hearing parents of children with HL expressed a desire to terminate a future pregnancy if the fetus were found to be affected [31]. Genetic counselors must be cautious in giving advice, providing detailed information and psychological support to advocate as wide a range of informed choices as possible. However, as a non-fatal monogenetic disease, the timing for HL prevention is more suited to a prospective technique such as PGT than either invasive or non-invasive prenatal testing methods.

PGT has been in development for almost 30 years, with the successful addition of techniques such as nested-PCR, multiple displacement amplification (MDA), MALBAC, array comparative genomic hybridization (CGH), SNP array and NGS [8, 32–34]. Routine PGT-M requires a complex process of preparatory work that incorporates linkage marker selection, primer design, development of amplification conditions, and single-cell validation [34]. These procedures are both time- and labor-intensive. Compare to STR marker, using SNP marker makes PGT-M procedure more efficient and simple. Our PGT procedure was developed base on MARSALA (mutated allele revealed by sequencing with aneuploidy and linkage analyses), which was first reported in 2015 [20] and had been proven to offer satisfied accuracy in linkage analysis [35]. We created a mini-SNP bank consisting of 90 SNPs within a 1 Mb region (upstream or downstream) of the *GJB2* and *SLC26A4* genes (Table S3 and Table S4). The frequencies of the major alleles in the SNPs were greater than 0.1 in the Asian population. This strategy allowed every case to provide sufficient heterozygous SNPs necessary for the linkage analysis. All families with *GJB2* and *SLC26A4* mutant genes were able to start the PGT process immediately, without any of the previously required preparatory work.

Whole-genome sequencing data provide euploid information simultaneously. Our results showed that 38.2% of the tested embryos exhibited aneuploidy, including monosomy, trisomy, or segmental CNVs, which may lead to implantation failure, miscarriage, or congenital disabilities. Thus, the simultaneous screening of CNVs, alongside the use of PGT-M, could increase clinical pregnancy rates to the level of 64.4% reported in this study, which is much higher than rates seen using PGT-M cycles without PGT-A reported by the ESHRE PGT consortium XIX-XX (24%) [36], similar to rates reported by groups in the USA and the UK (40.9–52.5%) [37, 38]. Previous studies indicated improvements in clinical outcome were obtained by trophectoderm biopsy, concurrent PGT-M, and PGT-A, and frozen embryo transfer. However, it remains to be seen whether different PGT-A methods (NGS, SNP array, or CGH array) can facilitate similar improvements in clinical outcomes.

**Fig. 3:**
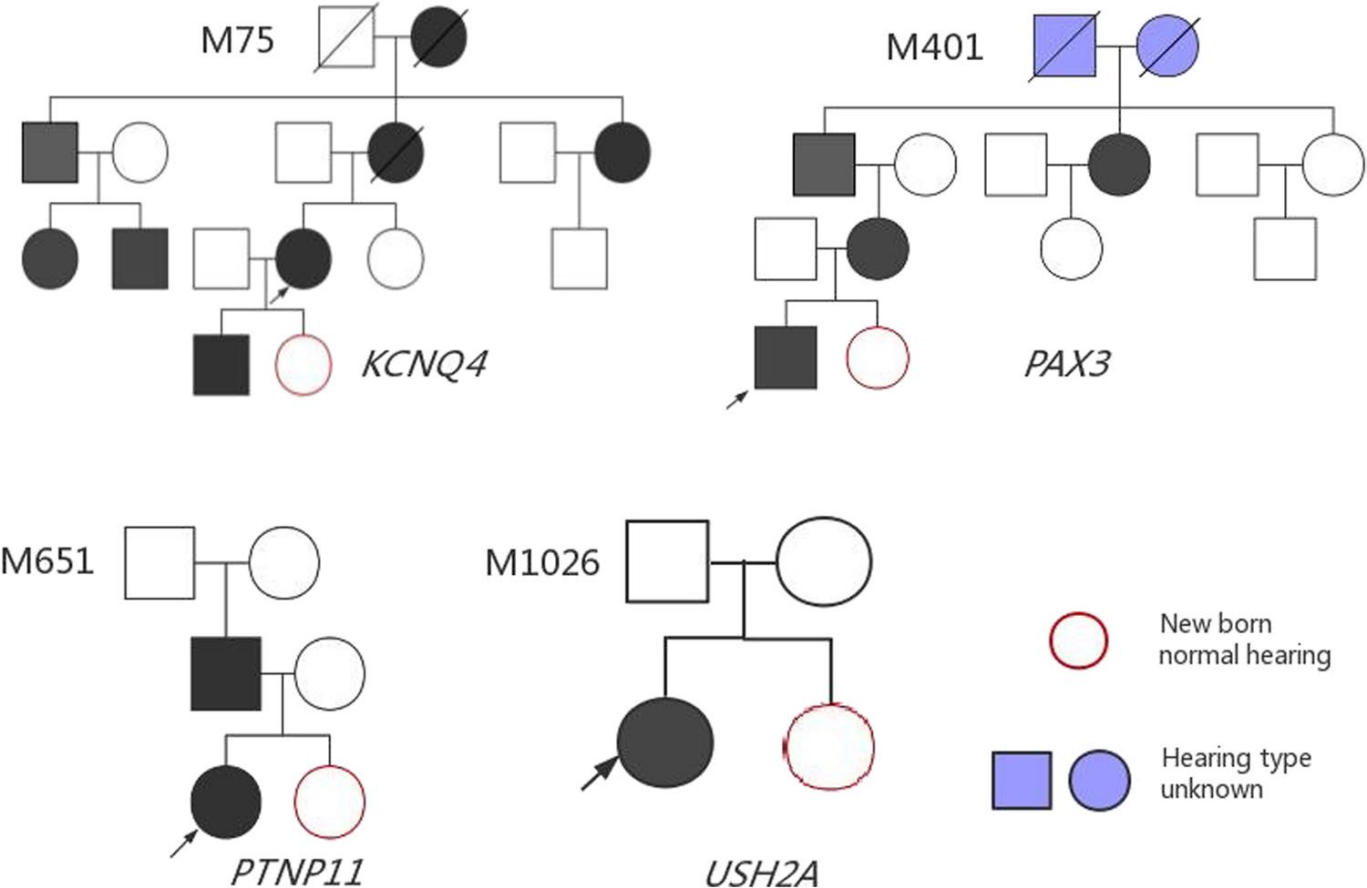
Pedigree chart of the four families carried rare HL gene variants

Hereditary HL is a typical monogenic disorder associated with high genetic heterogeneity, involving 124 genes that have been related to non-syndromic HL and more than 40 genes that have been related to syndromic HL (https://hereditaryhearingloss.org/). Identifying the pathogenic variants of rare HL genes is still the major challenge for clinical diagnosis and the clinical implementation of PGT. The present study enrolled 4 families with rare HL genes (*PTPN11*, *KCNQ4*, *PAX3*, and *USH2A*) into the cohort undergoing the PGT procedure. The variants were interpreted according to the ACMG guidelines and available databases (ClinVar and Deafness Variation Database). The pathogenicity of the variant *KCNQ4* c.733 G>A (p.Gly245Arg) was classified as uncertain and that of *PAX3* c.210 C>A (p.Cys70*) was classified as likely pathogenic. These classifications were upgraded to likely pathogenic and pathogenic through the co-segregation analyses of their pedigrees (Fig. 3). With the extensive application of multi-gene NGS panel and whole-genome sequencing, more HL patients will be offered access to an exact genetic diagnosis and PGT implementation for rare HL gene variants. Having said this, progress should be cautious, and a solid evaluation of the pathogenicity of variants is necessary.

Although the improvements made to the PGT procedure helped to reduce implementation difficulties and economical costs, more at-risk families still showed hesitance due to a lack of knowledge or fear of new technology, and/or worry about possible complications. As such, more cases need to be accumulated in order to objectively evaluate PGT and to streamline the process of introducing the possible intervention for at-risk families.

## Conclusions

In both the HL population and in hearing individuals at risk of giving birth to offspring with HL in China, there is a practical need for a PGT procedure to prevent babies from being born with HL. Considering the genetic heterogeneity of HL, defining the mutational spectrum through a large-scale molecular etiology study, identifying the target variants and a high-density set of SNP markers with high heterozygosity are key aims in the crusade for the prevention of hereditary HL. The modified MARSALA PGT procedure has the advantages of a simplified process, a satisfactory live birth rate, better generalizability, and has been validated as an effective and precise approach for preventing common hereditary HL in at-risk families, offering an alternative option to PND or to giving birth to children with HL who will require lifelong habilitation.

## Supplementary information

ESM 1 (DOCX 30.7 kb)

ESM 2 (DOCX 24 kb)

ESM 3 (DOCX 20 kb)

ESM 4 (DOCX 20 kb)
