# Correction to: Preimplantation genetic testing for hereditary hearing loss in Chinese population

**DOI:** 10.1007/s10815-023-02807-x

**Published:** 2023-04-24

**Authors:** Qingling Bi, Shasha Huang, Hui Wang, Xue Gao, Minyue Ma, Mingyu Han, Sijia Lu, Dongyang Kang, Aida Nourbakhsh, Denise Yan, Susan Blanton, Xuezhong Liu, Yongyi Yuan, Yuanqing Yao, Pu Dai

**Affiliations:** 1 College of Otolaryngology Head and Neck Surgery, Chinese PLA General Hospital, Chinese PLA Medical School, National Clinical Research Center for Otolaryngologic Diseases, Key Lab of Hearing Impairment Science of Ministry of Education, Key Lab of Hearing Impairment Prevention and Treatment of Beijing, #28 Fuxing Road, Beijing, 100853 China; 2 Departments of Otolaryngology Head & Neck Surgery, China-Japan Friendship Hospital, 2#Yinghua Road, Beijing, 100029 China; 3 Reproductive Center, Chinese PLA General Hospital, 28#Fuxing Road, Beijing, 100853 China; 4 Department of Otolaryngology, PLA Rocket Force Characteristic Medical Center, 16# XinWai Da Jie, Beijing, 100088 China; 5 Department of Clinical Research, Yikon Genomics, 1698 Wangyuan Road, Fengxian District, Shanghai, 201400 China; 6 Department of Otolaryngology, University of Miami Miller School of Medicine, Miami, FL 33136 USA

**Correction to: Journal of Assisted Reproduction and Genetics**

**https://doi.org/10.1007/s10815-023-02753-8**

The Funding information section was missing from this article and should have read 'This work is supported by the National Key Research and Development Program of China (NO.2022YFC2703602, 2016YFC1000706, 2018YFC1003100), National Natural Science Foundation of China (81870731, 81900953), Beijing Natural Science Foundation (7191011, 7192234) and Hainan Provincial Department of Science and Technology (819MS110).'

The original article has been corrected.

