# Supplementary material for: Preimplantation genetic testing for hereditary hearing loss in Chinese population
Source: J Assist Reprod Genet. 2023 Apr 5;40(7):1721–32. doi: 10.1007/s10815-023-02753-8 (PMC10352472; doi:10.1007/s10815-023-02753-8)
Supplement: Supplementary file 1 — (DOCX 30.7 kb) [file 10815_2023_2753_MOESM1_ESM.docx]

| Year | Type | Involved Genes | Single cell amplification method | PGT-M method | Linkage marker | Success Case No. | Region |
| --- | --- | --- | --- | --- | --- | --- | --- |
| 2021 | Non-syndromic  /Waardenburg Syndrome | *GJB2/ OTOF/SLC26A4 /MITF* | PCR | qPCR | Not mentioned | 11 | Taiwan [[1](#_ENREF_1)] |
| 2021 | Alport syndrome | *COL4A5* | WGA | NGS  SNP array(karyomapping) | SNP | 2 | China [[2](#_ENREF_2)] |
| 2019 | Usher syndrome | *USH2* | WGA (MDA) | NGS | SNP | 1 | China [[3](#_ENREF_3)] |
| 2018 | Non-syndromic | *GJB2* | PCR | Multi-fluorescent PCR | STR | 1 | Iran [[4](#_ENREF_4)] |
| 2018 | Non-syndromic | *SLC26A4* | WGA (MDA) | NGS | SNP | 1 | China [[5](#_ENREF_5)] |
| 2018 | Usher syndrome | *PCDH15* | PCR | Sanger sequencing | STR | 2 | Israel [[6](#_ENREF_6)] |
| 2011 | Non-syndromic | *GJB2* | PCR | RFLP | Not mentioned | 1 | Poland [[7](#_ENREF_7)] |
| 2010 | Non-syndromic | *SLC26A4* | WGA(GenomiPhi) | Primer extension mini-sequencing | STR | 1 | Taiwan [[8](#_ENREF_8)] |
| 2009 | Non-syndromic | *GJB2/GJB6* | PCR | Sanger sequencing | STR | 8 | Israel [[9](#_ENREF_9)] |

**Table S1: Previous reports applying PGT-M to hereditary hearing impairment.**

WGA: whole genome amplification; MDA: multiple displacement amplification; RFLP: restriction fragment length polymorphism; NGS: next generation sequencing; STR: short tandem repeat; SNP: single nucleotide polymo

1. Chen HL, Lin PH, Chiang YT, et al. Preimplantation Genetic Diagnosis in Hereditary Hearing Impairment. Diagnostics 2021;**11**(12) doi: 10.3390/diagnostics11122395published Online First: Epub Date]|. https://doi.org/DOI

2. Shi WH, Ye MJ, Chen SC, et al. Case Report: Preimplantation Genetic Testing and Pregnancy Outcomes in Women With Alport Syndrome. Frontiers in genetics 2021;**12**:633003 doi: 10.3389/fgene.2021.633003published Online First: Epub Date]|. https://doi.org/DOI

3. Luo H, Chen C, Yang Y, et al. Preimplantation genetic testing for a family with usher syndrome through targeted sequencing and haplotype analysis. BMC medical genomics 2019;**12**(1):157 doi: 10.1186/s12920-019-0600-xpublished Online First: Epub Date]|. https://doi.org/DOI

4. Karimi Yazdi A, Davoudi-Dehaghani E, Rabbani Anari M, et al. The first successful application of preimplantation genetic diagnosis for hearing loss in Iran. Cellular and molecular biology 2018;**64**(9):1718

5. Hao Y, Chen D, Zhang Z, et al. Successful preimplantation genetic diagnosis by targeted next-generation sequencing on an ion torrent personal genome machine platform. Oncology letters 2018;**15**(4):4296-302 doi: 10.3892/ol.2018.7876published Online First: Epub Date]|. https://doi.org/DOI

6. Yahalom C, Macarov M, Lazer-Derbeko G, et al. Preimplantation genetic diagnosis as a strategy to prevent having a child born with an heritable eye disease. Ophthalmic genetics 2018;**39**(4):450-56 doi: 10.1080/13816810.2018.1474368published Online First: Epub Date]|. https://doi.org/DOI

7. Liss J, Mirecka A, Kitowska K, et al. [Preimplantaion genetic diagnosis of hearing loss with 35delG mutation in GJB2 gene - preliminary report]. Otolaryngologia polska = The Polish otolaryngology 2011;**65**(6):443-6 doi: 10.1016/S0030-6657(11)70738-7published Online First: Epub Date]|. https://doi.org/DOI

8. Wu CC, Lin SY, Su YN, et al. Preimplantation genetic diagnosis (embryo screening) for enlarged vestibular aqueduct due to SLC26A4 mutation. Audiology & neuro-otology 2010;**15**(5):311-7 doi: 10.1159/000284349published Online First: Epub Date]|. https://doi.org/DOI

9. Altarescu G, Eldar-Geva T, Brooks B, et al. Preimplantation genetic diagnosis (PGD) for nonsyndromic deafness by polar body and blastomere biopsy. Journal of assisted reproduction and genetics 2009;**26**(7):391-7 doi: 10.1007/s10815-009-9335-5published Online First: Epub Date]|. https://doi.org/DOI
