# Supplementary material for: Preimplantation genetic testing for hereditary hearing loss in Chinese population
Source: J Assist Reprod Genet. 2023 Apr 5;40(7):1721–32. doi: 10.1007/s10815-023-02753-8 (PMC10352472; doi:10.1007/s10815-023-02753-8)
Supplement: Supplementary file 2 — (DOCX 24 kb) [file 10815_2023_2753_MOESM2_ESM.docx]

**Table S2. The genotype of transferable embryos and newborns**

| **Family**  **ID** | **Gene** | **Inheritance mode** | **Total Embryos** | **Transferable**  **(Wild type/carrier)** | **Implantation**  **Times**  **（Wild type/carrier）** | **Clinical**  **pregnancy** | **Outcome** |
| --- | --- | --- | --- | --- | --- | --- | --- |
| **F6711** | *SLC26A4* | AR | 17 | 11 (3/8) | 2 (3/1) | 1^st^ N,2^nd^Y | Wild type/carrier  (Twins) |
| **F6166** | *SLC26A4* | AR | 9 | 3 (1/2) | 2 (1/1) | 1^st^Y※  2^nd^Y | Carrier |
| **F3497** | *SLC26A4* | AR | 9 | 2 (0/2) | 1 (1/0) | Y | Carrier |
| **F1884** | *GJB2* | AR | 11 | 3 (2/1) | 2 (2/0) | 1^st^ N,2^nd^ N | Withdrew |
| **F7414** | *SLC26A4* | AR | 5 | 3 (1/2) | 1 (1/0) | Y | Wild type |
| **F8659** | *SLC26A4* | AR | 6 | 3 (0/3) | 1 (0/1) | Y | Carrier |
| **F8085** | *SLC26A4* | AR | 5 | 2 (0/2) | 2 (0/2) | 1^st^Y*,2^nd^ Y | Carrier |
| **F10284** | *SLC26A4* | AR | 7 | 0 | 0 | / | Withdrew |
| **F7047** | *GJB2* | AR | 3 | 1 (0/1) | 0 | / | Waiting for transfer |
| **F2530** | *SLC26A4* | AR | 9 | 6 (2/4) | 1 (1/0) | Y | Wild type |
| **F8241** | *SLC26A4* | AR | 13 | 3 (1/2) | 3 (1/2) | 1^st^ N,2^nd^ Y* 3^rd^ Y | Carrier |
| **F10449*** | *GJB2* | AR | 6 | 2 (0/2) | 2 (0/2) | 1^st^N,2^nd^ Y | Carrier |
| **F12131** | *GJB2* | AR | 6 | 3 (2/1) | 1 (1/0) | N | Waiting for transfer |
| **F12198** | *GJB2* | AR | 2 | 1 (0/1) | 1 (0/1) | Y | Carrier |
| **F8096** | *SLC26A4* | AR | 3 | 1 (0/1) | 1 (0/1) | Y | Carrier |
| **F932** | *SLC26A4* | AR | 16 | 4 (3/1) | 2 (2/0) | 1^st^ Y*2^nd^ Y | Wild type |
| **F12244** | *SLC26A4* | AR | 13 | 2 (1/1) | 1 (1/0) | Y | Wild type |
| **F9323** | *GJB2* | AR | 7 | 2 (0/2) | 1 (0/1) | N | Waiting for transfer |
| **F14118** | *SLC26A4* | AR | 10 | 3 (1/2) | 3 (1/2) | 01^st^ Y*2^nd^ N 3^rd^ N | Withdrew |
| **M464** | *SLC26A4* | AR | 4 | 1 (0/1) | 1 (0/1) | Y | Carrier |
| **F308** | *SLC26A4* | AR | 10 | 6 (4/2) | 1 (1/0) | Y | Wild type |
| **F12585** | *GJB2* | AR | 2 | 0 | 0 | / | Withdrew |
| **F3105** | *SLC26A4* | AR | 7 | 0 | 0 | / | Withdrew |
| **F6968** | *GJB2* | AR | 4 | 2 (1/1) | 1 (1/0) | N | Waiting for transfer |
| **F2242** | *SLC26A4* | AR | 4 | 1 (1/0) | 1 (1/0) | N | Withdrew |
| **F7105** | *SLC26A4* | AR | 2 | 1 (0/1) | 1 (0/1) | N | Withdrew |
| **F14171** | *GJB2* | AR | 6 | 1 (1/0) | 1 (1/0) | Y | Wild type |
| **F10717** | *GJB2* | AR | 8 | 2 (2/0) | 1 (1/0) | Y | Wild type |
| **F11595** | *SLC26A4* | AR | 12 | 4 (2/2) | 3 (2/1) | 1^st^ N,2^nd^ N 3^rd^ Y | Carrier |
| **F15108*** | *GJB2* | AR | 5 | 1 (0/1) | 1 (0/1) | Y | Carrier |
| **F14048** | *GJB2* | AR | 4 | 1 (0/1) | 1 (0/1) | Y | Carrier |
| **F13373** | *GJB2* | AR | 9 | 3 (1/2) | 1 (1/0) | Y | \| Wild type \| \| --- \| |
| **F15782** | *SLC26A4* | AR | 3 | 1 (0/1) | 1 (0/1) | Y | Carrier |
| **F11968** | *SLC26A4* | AR | 1 | 1 (0/1) | 1 (0/1) | N | Withdrew |
| **M759** | *GJB2* | AR | 9 | 3 (0/3) | 2 (0/2) | 1^st^N,2^nd^ Y | \| Carrier \| \| --- \| |
| **F8007** | *GJB2* | AR | 6 | 2 (1/1) | 2 (1/1) | 1^st^ N,2^nd^ Y | Carrier |
| **F15421*** | *GJB2* | AR | 4 | 3 (0/1) | 1 (0/1) | Y | Carrier |
| **F16250** | *SLC26A4* | AR | 8 | 1 (0/1) | 1 (0/1) | Y | Carrier |
| **F16178** | *SLC26A4* | AR | 11 | 5 (3/2) | 2 (2/0) | 1^st^N,2^nd^ Y | Wild type |
| **M1318** | *SLC26A4* | AR | 6 | 5 (2/3) | 1 (1/0) | N | Waiting for transfer |
| **M784** | *SLC26A4* | AR | 1 | 1 (1/0) | 1 (1/0) | Y | Wild type |
| **F17172** | *GJB2* | AR | 4 | 1 (1/0) | 1 (1/0) | Y | Wild type |
| **M855** | *SLC26A4* | AR | 10 | 1 (1/0) | 0 | - | Waiting for transfer |
| **M1026** | *USH2A* | AR | 10 | 5 (0/5) | 2 (0/2) | 1^st^ N,2^nd^ Y | Carrier |
| **Total** |  |  | 307 | 107 (40/67) | 55 (26/29) |  | Carrier 20  Wild type 12 |
| **M651** | *PTPN11* | AD | 11 | 3 (3/0) | 2 | 1^st^ N,2^nd^ Y | Wild type |
| **M75** | *KCNQ4* | AD | 20 | 2 (2/0) | 1 | Y | Wild type |
| **M401** | *PAX3* | AD | 2 | 2 (2/0) | 1 | Y | Wild type |
| **Total** |  |  | 43 | 7 | 4 | 3 | Wild type 3 |
