# Supplementary material for: Preimplantation genetic testing for hereditary hearing loss in Chinese population
Source: J Assist Reprod Genet. 2023 Apr 5;40(7):1721–32. doi: 10.1007/s10815-023-02753-8 (PMC10352472; doi:10.1007/s10815-023-02753-8)
Supplement: Supplementary file 3 — (DOCX 20 kb) [file 10815_2023_2753_MOESM3_ESM.docx]

**Table S3 SNPs and primers of *GJB2***

|  | rs ID | Forward Primer | Reverse Primer |
| --- | --- | --- | --- |
| YK-GJB2-SNP01 | rs7981756 | AACTGTGTTTGAGCCTAGGATGATCAAAC | TGTATACAAAAACTTTTGCCGGGATTTTG |
| YK-GJB2-SNP02 | rs9550642 | TGGCACACAGGGAACCTTGAGTAC | GTTCAGAGGACAGTTCCCTGTTGGTTC |
| YK-GJB2-SNP03 | rs9579857 | CCGCGGGCATTCCCGC | GGGTTTGAATGAGATGATGGAGAGC |
| YK-GJB2-SNP04 | rs2149695 | ACCTTGCTCATATTTGGGCAGAAGAG | TAGTTTCCTGAGGAAGATGATGACATTATTTAC |
| YK-GJB2-SNP05 | rs7989813 | CAATCTCTGCGGCCCAGGC | GTGAGTTGGTGGTGATGAGCTTCG |
| YK-GJB2-SNP06 | rs9552183 | AGAGCTGCTCTGACACTTTGGAAATACTAGAG | CTGTTGCCCCTAGGTGAGCAGAGAC |
| YK-GJB2-SNP07 | rs3936015 | TTAGCAACCCCAGCTCTTCCTTCTC | GTTTCCCTGTGCTGGGTCACCC |
| YK-GJB2-SNP08 | rs2872488 | AGTGCTGGGATTATAAGCGTGAGCC | AGCTGATATATTACGAATCTTCCAAATTACTTATATGAG |
| YK-GJB2-SNP09 | rs7336556 | TCTGATGGTTTTATAAAGCGGAGTTTCC | ATGGAGTCATAGTTCACGCGGCTG |
| YK-GJB2-SNP10 | rs9509271 | AAGAAATGGGGAGTTTGGAAATAGAGTTTC | TTAACCATTTTTGTGTGTACAGTTCAGTACTGT |
| YK-GJB2-SNP11 | rs6490593 | TGTGCCACTTCACTCTAGCCTGAGC | CCTCTCTTCTTTTCTCTCTCCCTTCCTC |
| YK-GJB2-SNP12 | rs9550662 | CCGTCTCCTGCCCGCCC | CGCCCGCGTTTCTGAGTAGACA |
| YK-GJB2-SNP13 | rs9552248 | CTTTGCTTTAGATGAAGTGTCCTTCTGCTG | GATGGGCAAGCTACTCACAATGATTTA |
| YK-GJB2-SNP14 | rs3825444 | CATTTTTATAATGACAAGTTAAAGGTTAAAGCATTGT | CATTAAGTTATATCAGGGTAGTAATGGCCTTCAG |
| YK-GJB2-SNP15 | rs9506528 | AGAAATATGTTTTTAAAATTACACGCCATGAC | TTTCCTTAGTTTGTTAGTATGGTGAATTACATTG |
| YK-GJB2-SNP16 | rs12873775 | AGGAAGTGGGGAGAACATTATCTACTAAAGAATATG | ATGCAATGGAACCACCCCATGG |
| YK-GJB2-SNP17 | rs1052209 | TGTTCTGTTTTGTTCATTTAATTTCTCTGAAAC | CAGTCTCAAATACATCAGTTGCTTGACTG |
| YK-GJB2-SNP18 | rs4769124 | TCATTTAAAGTTCAAAAACAGGCTAGATTGAC | TGTGGTATTATTATTTATGTTTCTGAATACATTACCTTC |
| YK-GJB2-SNP19 | rs1411038 | TAATAGTTAACAGTATTTATATTCTCTGCAGATAAACTGATG | GTTCTGTCAATTTCTCAACCTTTAACAAAATTC |
| YK-GJB2-SNP20 | rs2149769 | GAGGAAAACCAAAACTAGACCACATTCC | AATGAATTCTCTGCACATACTGGATGTTTAC |
| YK-GJB2-SNP21 | rs9315465 | CCGGTCTGTGCAGGATGAGATTCC | CTCCAAACATCTGCCAACACTGCAC |
| YK-GJB2-SNP22 | rs11147641 | AGCACTCCTGGGGTTTTGGCC | GTCTTTAAATGTAAATACTGCACTAGAAACAGCTAATC |
| YK-GJB2-SNP23 | rs12867384 | CCAGCTCGGGCAGTGACTACG | TTGAGGGCTGCCTCCGTGATG |
| YK-GJB2-SNP24 | rs1887895 | TTGGCATGGAGAAGGTGACAGACAC | GTGGCCAAGTGTAGTGGAACAAATTGTAC |
| YK-GJB2-SNP25 | rs59381570 | GCTTCCATTTACAAGATGGAAAGCTTCT | CATAGGATTTATCACTTAACCATTGTTAAGCG |
| YK-GJB2-SNP26 | rs4769153 | GCGGGAGAGGAGGGTGGAGG | TAAAAGTCCTAATCCCTTGATTAAAAACCTTC |
| YK-GJB2-SNP27 | rs2016319 | GAATTGTAGCCCCTGTAATTCCCACG | TGAGACTTATTCACTATTATGAGAACAGCATAGG |
| YK-GJB2-SNP28 | rs945373 | TGCAGGCCCATAGGGAGGG | GTCTGAATAAGCCTTTTTATTCTATGTGCTTCC |
| YK-GJB2-SNP29 | rs9550636 | GGGGTCTCCACACTCACCCCTG | ATGGCAGCGAGTTGCAGGTCA |
| YK-GJB2-SNP30 | rs7994377 | ATTTTATAATCAAACCAAAGTTTCAAAATTAGAAAAG | TAATTTTTAAAATACTATGTACTTTTTTTCTAATTATGAAAACTAGTC |
| YK-GJB2-SNP31 | rs9316031 | GGCAGGGGATGGAAGGGAGG | AATACAACAGAAGCATTGTGAGAATTCAATG |
| YK-GJB2-SNP32 | rs9550685 | AGGCGTTAGTCACCAGCCTAAAGAGAC | GCTCAGCAGCCTCACTGCTGTAGTC |
| YK-GJB2-SNP33 | rs80292533 | GCTATGCGATGAGATCACGTCTACG | TTTTTTTAATGGGCGCCAAAAGC |
| YK-GJB2-SNP34 | rs9552325 | GATAAACTGAGGAAAGGTGTGTAAATTGGTAC | TGGAAGTGGAATCTTGGTTGTAGGATTTC |
| YK-GJB2-SNP35 | rs9550698 | GGCTGGAAAGCTCTCTCTGGAATCTG | AAGTGATCTGCCTGCCTCGGC |
| YK-GJB2-SNP36 | rs9319266 | CAGACAAGTTTTACTGGAGAAATGTCCAAAC | AGGCATTTCTTGAGATTGTAGGTTTCTAGAAG |
| YK-GJB2-SNP37 | rs7332299 | GCTGGGAAAACTGGATATCCATAGGC | TGTTGGGTTTTATGTTTAAGTCTTTAATCCATTC |
| YK-GJB2-SNP38 | rs1854787 | TAAAAACTTAAAAAAAAGTGGCTACCTTTGG | CCCAATAGGTTTGTTAGTCATAGTGTTAGATCTCC |
| YK-GJB2-SNP39 | rs7985257 | ACTATAAGCAATATTTTCTGAATCTTGCAATTAAAAAAATAC | TTTAGGAAAAATTGAATTATTCGTTCATTGAAAG |
| YK-GJB2-SNP40 | rs8000138 | GAGTAAGGTCAAGGTGGAGAAAAAAGAGAC | TAAGACATAGTTAATAATGCAGAATAAATATACAAAAACAAG |
| YK-GJB2-SNP41 | rs7327207 | TTTGGCTTGTGAGCGTTAGTTTGTCA | CTGAATTGAGTGATACAAAACTCTGCCTG |
| YK-GJB2-SNP42 | rs9552058 | CCTTTTTTTTTGAAGGTTGATAGTTTTCTTG | TGGCACAGAGTTCTTATCACTTCCAT |
| YK-GJB2-SNP43 | rs4769950 | TCGCTTCCACCTTTTTGCTGTTG | CACTCCCAGTGGGCCAGGCT |
| YK-GJB2-SNP44 | rs4769086 | CTGGACAATGCCACTGGCGTTTC | CACGATAGGCATTTGGGGTGGG |
| YK-GJB2-SNP45 | rs767846 | CCTGCTCACAGCTGCTGGCC | CTGCCTGAAGGCCTGGGGG |
| YK-GJB2-SNP46 | rs1599138 | GAAGGACACTCCTAATTGAACTGACAATAATTTC | AACTTCTATGCTGGTATCTGCACTTCCTG |
| YK-GJB2-SNP47 | rs7329320 | GGGCTGCAGAATGCTCCAGC | CCGGCTGGTTTATGCTCTTCCTG |
| YK-GJB2-SNP48 | rs7992032 | CACTCTTGTCGCTCAGGCTACAGTG | AACCCCAGCTACTCGGGAGACTG |
| YK-GJB2-SNP49 | rs9551930 | AGAGGTTGTGGTGAGCCAAGATCAC | ATCTTGCCAGCAATCCCGTATTTC |
| YK-GJB2-SNP50 | rs60135215 | GTCATGGAAATTAGCCGAAGCAGGTAC | AAACCTTCACCGATATCATCGGAACC |
| YK-GJB2-SNP51 | rs9512695 | TAAAGTTTTGTTTGTCGCGCTGCTC | GCTCACCCCTGTCCCCGAGG |
| YK-GJB2-SNP52 | rs1599138 | TGAAGGACACTCCTAATTGAACTGACAAT | AACTTCTATGCTGGTATCTGCACTTCCTG |
| YK-GJB2-SNP53 | rs7981716 | CACTCTGAAGCAAAGAAAAGCAGACAAAG | ACAACTTAGAACAGTATCTTGAGTCCTATTGCG |
| YK-GJB2-SNP54 | rs61945043 | GTTTTCCCATGTTGGACAGGCTG | GCCAGTGGGCGCAGTGGC |
| YK-GJB2-SNP55 | rs9511774 | TCCACTGTGTAGGGTGGCTATCCACTAC | AAATGTAATGTGTATGAGCTTCTCATAGAAATCC |
| YK-GJB2-SNP56 | rs7330385 | GCGCCCAACAAGTCCTGACAG | TGTGGAACATTTCACCCACAGTGC |
| YK-GJB2-SNP57 | rs9511363 | AGCTGCATTCGAGGGCAGGC | GCCCACCTGCCATGGGAGTC |
| YK-GJB2-SNP58 | rs995813 | GTCAGCATGGGGGATCTGAGGAAG | AAAAGTATGCACTTTATGGTCTTCACGC |
| YK-GJB2-SNP59 | rs1889574 | ACACACAGGGCTTCATGGATTCACA | TCAGAGGCCAAGTGGAAGTACAAGGT |
| YK-GJB2-SNP60 | rs1937743 | GGTGCACCCCACTGGGGGT | GTCCCTGGGACCACCCCCAC |
| YK-GJB2-SNP61 | rs76386906 | TTCTGCACAGCATCCTGCCCG | GTGTTGCATTGTTTTTTGCAGTAAGAAC |
| YK-GJB2-SNP62 | rs7325766 | CTTCACAGGGGCATACGAAGGTCA | CCCCCTCGCTCTAACTTAAGCCAGT |
| YK-GJB2-SNP63 | rs4769988 | TAGGCAGGCCCTATGCACCTGG | TCCCACCCAAATCCAAATGCAG |
| YK-GJB2-SNP64 | rs1007767 | TGGAAGCCTTCCCTGTCCCTGTC | GACTGTGGCCACGGATCTGAGG |
| YK-GJB2-SNP65 | rs9509138 | CCTCACTGGGTCTCCAGCCTGC | ACAGAATTAGAAGGAGACTGATTTTTTAGGAATTG |
| YK-GJB2-SNP66 | rs1335870 | TAGGGGAAATAATTAGCCCTTTTAGGACATTAG | ACTTGACTTAATGAGATCCCTTTTCTGGAG |
| YK-GJB2-SNP68 | rs7332444 | TCTTCCTCATCCACTTCTTTTTTACTAATTCC | TGCACAAGAAAAATGTGAAAATTCAGGAC |
| YK-GJB2-SNP69 | rs9509208 | AAGCGGCACCTGGGGCTTTG | GCAGGGGGTTGGGGGAATCT |
| YK-GJB2-SNP70 | rs9552178 | ACAGGCTCTGACATTCTTCTGTGTCAG | AGTGATCCCTCCACCTCGACCTC |
| YK-GJB2-SNP71 | rs9552180 | TTACCTCCACATTCTAAATGGCTCTTGC | ATGATGTCACAGGACTTGGGCAGTAG |
| YK-GJB2-SNP72 | rs9552189 | TACAAGCTCGTGTAACTAAGAAGAAACGG | TGCCTGCACGGGTGGGAG |
| YK-GJB2-SNP73 | rs9315651 | ATTAATCTTTTCAAAATGCAGACAATAAAGTAAGC | GCTGGTTATTATCCTTATTTGGAGGGTG |
| YK-GJB2-SNP75 | rs9509279 | CTGCCCACAACCTCTGACTAAGTGG | GCACAGTCAAAACCCTAACACAGGG |
| YK-GJB2-SNP76 | rs7328681 | TGGGCATTCTTCACTGATATCTGCTG | TGAAGTCTTGGAGGCTAGAGGAAAATG |
| YK-GJB2-SNP77 | rs1360786 | CTTCCTGGAATTGAATCTCTCTCTCCAG | TCTATCCTCTTAGTCCTGTTGCCCTAGAG |
| YK-GJB2-SNP78 | rs4770464 | TTTTTTTTGGACACTTTCTCCAGGAGG | TGTCTGTGATGGGGACTGGGAGAAG |
| YK-GJB2-SNP80 | rs6490946 | GGAAGAAAACAGGAAAAAAATTTAAGAGAGAGAG | TCGCCTGAGAAGCACAGGACGA |
| YK-GJB2-SNP81 | rs17063328 | CGGAGGGAACAGGGCCAGG | TTCCTCCCTCCCACATCTCTGAGAG |
| YK-GJB2-SNP82 | rs73433998 | TTGAACCCAGGAGGTGGAGGC | TCATACTTTTCTCCTTTCTTCTTTTTTGAGATG |
| YK-GJB2-SNP83 | rs504544 | GTCACTGCTGATGGTCAAGGAGGC | CCCTGGCAGGGGAACAGATAAGG |
| YK-GJB2-SNP84 | rs521244 | GGGAACCACACCTTCATCTCCCG | GTGGGTAGGGAAGGACCATCAGGT |
| YK-GJB2-SNP85 | rs9551322 | ATTCTAAACAATATAAAAAGGACTCCTCCCTACTC | ATCAGCCTGAAATTTTCTTTTTTTGTTGTG |
| YK-GJB2-SNP87 | rs9507826 | AAAAAGATTGTATATTCTCCCGGAAATAATTGTC | TTCTTGTACATCTTCATCAGAGCTCTTGGC |
| YK-GJB2-SNP88 | rs56097819 | TTGGGCAGCAGTGCCTTCAAG | ATTATAAGAGGTAAAGTGAAAAACAGAGAATCTGT |
| YK-GJB2-SNP89 | rs77910629 | GTTCCTGTGTGAGGATATGTAAAAAGACAGG | GAATTGGGCAGGAGGGCCA |
| YK-GJB2-SNP90 | rs73154200 | GGGACATAACACTAGCTAAGGCCAAAAG | TTTTCAAGACTGTTTTGGCTATTTGGAG |
