# Supplementary material for: Preimplantation genetic testing for hereditary hearing loss in Chinese population
Source: J Assist Reprod Genet. 2023 Apr 5;40(7):1721–32. doi: 10.1007/s10815-023-02753-8 (PMC10352472; doi:10.1007/s10815-023-02753-8)
Supplement: Supplementary file 4 — (DOCX 20 kb) [file 10815_2023_2753_MOESM4_ESM.docx]

**Table S4 SNPs and primers of *SLC26A4***

|  | rs ID | Forward Primer | Reverse Primer |
| --- | --- | --- | --- |
| YK-SLC26A4-SNP01 | rs7786720 | GTTAACATTATGTTATCTGCTGGTTAGCTCTGTG | GAACTGGTTTGTTACAGCAGTGTATCTTATCTG |
| YK-SLC26A4-SNP02 | rs6946733 | CTTCCAAGGCTTATTCTTATTCATTTGCTTA | GATGCAGTCCTGGGCCAATTTATG |
| YK-SLC26A4-SNP03 | rs2536505 | AACAGTACCTCATGGTCCCATTTTTAAAC | CTCTCTGTCTTAAGGATGTACTGATAAGAAGGG |
| YK-SLC26A4-SNP04 | rs717099 | GAGTTTAACAGTTTGATTAAAATGTGTGTGGG | GGCCAACTTTCCAAATTTTACAAAAAC |
| YK-SLC26A4-SNP05 | rs2302453 | GTAATTGATAGGTAAGTTTTGCCCAACCTTAC | GCGGCAATAACAACTATCCTATATTAACCTG |
| YK-SLC26A4-SNP06 | rs4730221 | AGCGGCCCGCGCCTG | TCCAGTCTGCCCAATCCCCC |
| YK-SLC26A4-SNP07 | rs2158347 | CCAACTTGTTAAGATGAGAAAAATGAACG | GCAATGTCTGTGTGGATTATTTTTCCC |
| YK-SLC26A4-SNP08 | rs7794437 | GTGGAAAATTTACAACGTTAAATGTTTATTTGTG | CTTTCTGTTTATCTTTGGATTTGTGAGTTTTC |
| YK-SLC26A4-SNP09 | rs6966616 | GTTTATTATGGTATTTCAACCGGGAATGG | CTAATTTTAGACAGTGTGTCAAAATCTGATTGTC |
| YK-SLC26A4-SNP10 | rs10260250 | GGCAGCAACACAAAGTTATGGAATCC | GGCTTACAGAGTTAGAAGTTTGGAAGAGATTG |
| YK-SLC26A4-SNP11 | rs2107763 | CTCCCACTGTATGTAAATTATAAAGGTTTTAACC | GAGGCCACCTGCAGCTAAAGCTG |
| YK-SLC26A4-SNP12 | rs6979121 | GGTATTATGAGAGTCACATATCCACAGTCAGAAG | CTGAGTTAACTTAAAGCCCTTAAAATTTGCAG |
| YK-SLC26A4-SNP13 | rs10276321 | CCACTGTTGGTTTGTCCCTGAAGTC | AGTAGTCTACTTTGATGCCCTGGATGTTG |
| YK-SLC26A4-SNP14 | rs2520279 | GCCAACAATGGCAAATCAAGTACTTCTC | ATAGGCTTTGAGATGGGGAGATTATTCTG |
| YK-SLC26A4-SNP15 | rs2520257 | ATGATGTCTTTGCAAATATTAAATCACACATC | AAGATATAGTTCATGGTGGAATGATCTCAAAG |
| YK-SLC26A4-SNP16 | rs13224313 | GTAATTCAAGGCAACCACAATTAAGGG | TTACTTTAGATCTGATAAAATGTTAAACATTTGTTTG |
| YK-SLC26A4-SNP17 | rs2269778 | CATCTTCCTTACCACTTACCTCTCTAATCATC | GCACATGGCAGACATAAGGTAATAGTTGG |
| YK-SLC26A4-SNP18 | rs989960 | GCTCACACCTGTGCTAACTGCGTAAC | CACATTGCAAAGTTACAGCCCATACTAAAC |
| YK-SLC26A4-SNP19 | rs2108227 | GCCTGCAAAGATGTCCATGTTCTAATTC | CATTTCAAGATCCTTAACTTAAACACATTTGC |
| YK-SLC26A4-SNP20 | rs10272963 | GTGGTTTAAAACAACACAGATATATTATCTTACAGC | AGCCTTCAGAGAGGAACATAGCCATG |
| YK-SLC26A4-SNP21 | rs7811034 | CAGCATGTAATCAACATAAATGATGATTAAAATG | CAACACATCATCAGTTGGAAGCCTTG |
| YK-SLC26A4-SNP22 | rs17412104 | GCTTTACTTTGAATACAAATTAAACTTTGCATTATAG | CAGGGAAATCTATTTCAGGTTAGCAAGC |
| YK-SLC26A4-SNP23 | rs1131398 | GTATATGTAGGTGTGTTCCCATTCATGTAATG | AGAAGGCAATTAAACAAGCAGATGAAGAC |
| YK-SLC26A4-SNP24 | rs2072208 | GAACTGTTTACCTGAGAAGGTCTCCATTG | CCAAGCCCTGTTGCAAAAGAGC |
| YK-SLC26A4-SNP25 | rs1544474 | CATGCTGTACTGGGTCTATGGCACG | CCAACACCATAGTAGTCTGCTGGGAAG |
| YK-SLC26A4-SNP26 | rs12670994 | CAGACATAACTTTGATTTTGATTGGGAAAAC | GTAATTTTATCTAGTCTCCTATTGATGGTCATTTG |
| YK-SLC26A4-SNP27 | rs1990158 | TCACAATCTCTTCTGGCACTCTTCCTC | AGCACATAAATGGAGAACTGGAAAACG |
| YK-SLC26A4-SNP28 | rs390547 | CCTTCATTTGATAAATGTTTAGTAACAATTATGTG | CATTTCTGCCTCAGATGCGAGATTC |
| YK-SLC26A4-SNP29 | rs401487 | CAATCCTCCACTCTCCAAAATAGGGTAG | TGCCTGGATCCTACCCACTGTCTG |
| YK-SLC26A4-SNP30 | rs760355 | CCAGGTCATTTTCTCTACTCCTCCATTC | GGAGGTATAAGGAGAGAGTCAGCCACAC |
| YK-SLC26A4-SNP31 | rs17153388 | TGCACATTCTGGCTCTGTCACTTACG | GGCAAAAGAAAATAAGTGATTAAATCACATAGC |
| YK-SLC26A4-SNP32 | rs2107910 | TCTACAGTAATCAAGACAGTGTGGTATTGGC | CCCTTGTTGGAAATCAATTGACAGTAAG |
| YK-SLC26A4-SNP33 | rs17153394 | GTCATCTATCAGCACTAATGACATCTCTATACATG | CAGAAGGCTGTCTTTTAGTGACAGAGAGAG |
| YK-SLC26A4-SNP34 | rs6466131 | CTGTAACCTAACTGGATGTATCAACAGACTGTAAC | GTTACAGCTTGATGTTTGTCTTATTTGAACG |
| YK-SLC26A4-SNP35 | rs2132462 | GCACATCATCATTGATTTCCAACTTAGACTAG | CTGTGCTAATTACTAGAGATGCAGAGATGAAC |
| YK-SLC26A4-SNP36 | rs10268373 | GGTGAAAACCCTGGTTAGTAAGTGATTTTG | GTCCTAGGACACGTGAAGTTAAATGCTG |
| YK-SLC26A4-SNP37 | rs2028009 | TCTTCCACCACCACCTCCTGTCC | ACTCCCGATTATTTCATCTGATGCAAC |
| YK-SLC26A4-SNP38 | rs10274710 | TCAATTGGACTTCCAGGGTATCATGG | CCCAATGTTTGTTTATCACCATAGATATTTTG |
| YK-SLC26A4-SNP39 | rs4727663 | TGGAGAGCAAAACAAAATGGCATTC | TGACAAAGAGACACCTTGTGAGATTTGTG |
| YK-SLC26A4-SNP40 | rs849380 | TCACACAGACCTTCCACCAGTCTCC | CCCCATCACTATGAAAGTTTACCATAATAAAAG |
| YK-SLC26A4-SNP41 | rs1526083 | CAATGGAAGCCTTCTCAAAAGGAATTG | AGGCAGCTTCATCATATCTACTTTTGCC |
| YK-SLC26A4-SNP42 | rs12536620 | GTTTATTGATTCCAGTATGATGTTCAAAAGC | CCTTTTTAATTCAGTTTCTTTCCCAAGAG |
| YK-SLC26A4-SNP43 | rs11763202 | AGCACTTTTGGGCTCCAGCCC | CAAGCCATATGTAGATGGCTAAGCTGAG |
| YK-SLC26A4-SNP44 | rs10953524 | ACATGCACGAGACACCACGCC | TGAGTTACTAACATTTGCAACTTGAGATATATCC |
| YK-SLC26A4-SNP45 | rs12530679 | GAGATGTTTGCTTCTTCACTTGTAGAATAATATAATG | CTACCACATCTGGCATACTGAGGATCATC |
| YK-SLC26A4-SNP46 | rs2072546 | TACTGGATGGGCGGACAACAATG | CATGAGCGAAGGGAAAGCTGAGAC |
| YK-SLC26A4-SNP47 | rs1035204 | GTTGAAGATCAGCCTGATTGGAATAGC | CAGACAGTTGTATCCTGTGATCGCTCC |
| YK-SLC26A4-SNP48 | rs10262724 | ATGGCAGAGATCAGGCCTTGCTC | GAAGCAATAGAGCTTGGGCCACTG |
| YK-SLC26A4-SNP49 | rs2111201 | TTCCTGGATCTTGGAGTTCCAATCTG | GCAGCCAAAGAAGTCTCCATAGAGATG |
| YK-SLC26A4-SNP50 | rs17155518 | GGAGATTGGCTGTTATCCTTGGGC | TGCCTTTGATCCTCTCCTGCAGC |
| YK-SLC26A4-SNP51 | rs13244715 | TGCTGAAAATTAACAGCCCTGAAAAAG | TCTTATATCCCCCACCCTGCAACC |
| YK-SLC26A4-SNP52 | rs7783893 | ATGTTACATGGGAAGTGCCATGGC | CTCTCTATTGATGGGAAGGACATTTAAAAAC |
| YK-SLC26A4-SNP53 | rs12333431 | GCTTGGCAGAAACAGGAATGAAATTG | GGATCCACTGCTGTAAACATAAATTGTAAAG |
| YK-SLC26A4-SNP54 | rs1859768 | CTCACATGGATTTCACACCCCTATCC | CCTAGAGGAGGTGTCCCCTAAAATGG |
| YK-SLC26A4-SNP55 | rs2300043 | CATAAAGATACATTTTAAAGTATAATGGGGTTTCTC | GCCTGGCACTGTTCTTAGTAGGACTTTC |
| YK-SLC26A4-SNP56 | rs13221639 | GTACGCTAATATTCATAGCAACATTATTAATAATAGTCTC | CAGTGGTATTCCCTGGTATGTAGATACCAC |
| YK-SLC26A4-SNP57 | rs3763462 | GGTTCCTGAACCAGCATGATATCTGG | AGGTACCACCTGAATGGCAGTTGG |
| YK-SLC26A4-SNP58 | rs2396001 | GAAGTTATATAAGGTCAATAAGAAAAATCAGTTGTATTTC | AGCGTTGACCTTGACCCTTTGC |
| YK-SLC26A4-SNP59 | rs194585 | GCCATAGTTAAGAGCAACAGAAAGAGCTG | ATAGAATTTATGTGCCTAGAAATTTCATGCAG |
| YK-SLC26A4-SNP60 | rs40856 | CACAAGAGTGAGAAGGTCAGCCACG | CTTCATTATTTGTAGAATTTTAAAAATATATTACGGTTG |
| YK-SLC26A4-SNP61 | rs917902 | CCTCGAAGCATCTTTTAGAGATTGACTTAG | CCCTTTCTTCTCCTATGGGATTTACC |
| YK-SLC26A4-SNP62 | rs577004 | CAGTACTCCTTCAGCTGCACCTGAGTAAG | AAACTGCCTCCTTATGCAACCGAG |
| YK-SLC26A4-SNP63 | rs997438 | GCATTTGGGGAGTAGAAATTGAGAATAGG | GGTGTTGTTATGAAGGCAGTTTAGTCACC |
| YK-SLC26A4-SNP64 | rs12673582 | CAGGGTGGAGTCCACAGGGCTG | ATGTGGCAGAAGAGCTGGTGTGTTG |
| YK-SLC26A4-SNP65 | rs4730123 | GTCTTGCTAAACAGTGAGGGAACACTCTC | AAAATACTCTATTGCTTTGATGGGTTGGG |
| YK-SLC26A4-SNP66 | rs2072161 | CTACTCAGCCTTGTTTGCAGCCAC | TCTCTGATACACCAGAGCTGGATTAGTTATATG |
| YK-SLC26A4-SNP67 | rs10270308 | GTGTTTGATGCTGTCTCTGTCTTCTCCTTAG | GCATTCTTACAGTGGTATTGCCACTTAATTAC |
| YK-SLC26A4-SNP68 | rs367311 | ATAGGTGTGTGGGGAGAGTGAAGAGAC | AGACCTCGAAATCACCCTGCAGG |
| YK-SLC26A4-SNP69 | rs73414213 | TCTATGGAAGCTGGAATCCACTCTCC | GGTATTGGACTGAAAACAAACTGCTCG |
| YK-SLC26A4-SNP70 | rs887882 | GGAATTAAGGAAAAGGGCACAGATCTG | GCACTACAGAGTCTGAATGCTGGTTTG |
| YK-SLC26A4-SNP71 | rs6973049 | AGTTGGCAGTCCTCTGCCCAGG | GGCAACCAAAGACTGTGTGTCCATC |
| YK-SLC26A4-SNP72 | rs10273098 | GACCTCGTGCTGCTTAGTAACATCTGATC | ACAGCACAGAAAGGCCAGCGC |
| YK-SLC26A4-SNP73 | rs2392934 | AGGCTTAAGGTTTTAATCATAAATTAAAGTTCAAG | CCTCACACTTCTGTCAAAACATTGTGTG |
| YK-SLC26A4-SNP74 | rs2188420 | GGAATATTCCCATGGTGACCCTTACTG | GGAATCTTTTTACTCTAAGGCTGGCAG |
| YK-SLC26A4-SNP75 | rs7780542 | GCTCACATACGATAGCACTTAAGTGGTTTTC | AGCCAGACTCTGGGGAAATGTTTTC |
| YK-SLC26A4-SNP76 | rs12667481 | AGTTCTGTAAGAAAGTCACATAATGACAAATACCTAG | CTTTCCCCTCTGAACTTCCTTTCCTTC |
| YK-SLC26A4-SNP77 | rs2237681 | GGAGATGAGAGAAGATAAATGACTTTCTCAAC | CCTGACATTGAATATGTAGCAGAGAAGAAGAC |
| YK-SLC26A4-SNP78 | rs11982404 | TTCCTAAAGGGTAGGATGAAATAGTTCAGC | CCTAGGCAACAGGGGTAGATGCTG |
| YK-SLC26A4-SNP79 | rs1017057 | GGAGAGATGGTGAATTGGAAAGACATG | AGACGTTGTCTGCAGCACTTGTGTG |
| YK-SLC26A4-SNP80 | rs73193703 | CCACCACACCACGAAAGTAGTAAGTTCC | TGGAGGCTTCCTCAGTGTTGAAGG |
| YK-SLC26A4-SNP81 | rs7792061 | CTCAGTGCGCAGCCCATGTTG | CGAAGGCATTTTCTAAATGACAGGATTC |
| YK-SLC26A4-SNP82 | rs41281051 | TGCTGGGGATGGGGGAAAAG | CCAGTGATGGAACTGCTAAGTCAAAGTAC |
| YK-SLC26A4-SNP83 | rs58442437 | GAGGAAACACTCCAACATGATTTGGC | CTCATCATTTCCTTTGTGCTGGTCC |
| YK-SLC26A4-SNP84 | rs424978 | GTCTCACTAGTGTCATTTTGTAAATTTCCTCAG | AAGCTGGCCCTGTCCATTTGC |
| YK-SLC26A4-SNP85 | rs409815 | GGGAGTAGTATATAGGATTTTTTAGGAGCATAAGAG | CCATTTAGAGCATTTTTGGGAAAATCC |
| YK-SLC26A4-SNP86 | rs13242382 | TCTGTAAAGACACCTGCTTTTCTTCAACAG | CTAAACCTAATCCTGCTCCTCCTTACCAC |
| YK-SLC26A4-SNP87 | rs10953563 | TTGGGCTAGACAGATTAATTTTCTTGTTTAAG | AACATGTAAAAAAGCATACTGTGTTGGGAG |
| YK-SLC26A4-SNP88 | rs2284285 | ACAGAAGCTTCGCCAATGTCAGG | CTTGAAAGCCTGGCTCAGGCTTC |
| YK-SLC26A4-SNP89 | rs6948554 | AGTATAATCTTTGAAGAGACTTACATACACTGACCTG | ACACCTACCTCTTGGCACGACTATAAGG |
| YK-SLC26A4-SNP90 | rs11534043 | AAACCCTAAGACCACTGGAGTCAAATACAC | TTACTCCCTGTTAGGTGCTCTAAGCACC |
